# Phylogenetic diversity of culturable fungi in the Heshang Cave, central China

**DOI:** 10.3389/fmicb.2015.01158

**Published:** 2015-10-21

**Authors:** Baiying Man, Hongmei Wang, Xing Xiang, Ruicheng Wang, Yuan Yun, Linfeng Gong

**Affiliations:** ^1^State Key Laboratory of Biogeology and Environmental Geology, China University of GeosciencesWuhan, China; ^2^Laboratory of Basin Hydrology and Wetland Eco-restoration, China University of GeosciencesWuhan, China; ^3^Key Laboratory of Marine Biogenetic Resources, Third Institute of Oceanography, State Oceanic AdministrationXiamen, China

**Keywords:** fungal diversity, the Heshang Cave, culturable fungi, ITS sequences

## Abstract

Caves are nutrient-limited and dark subterranean ecosystems. To date, attention has been focused on geological research of caves in China, whilst indigenous microbial diversity has been insufficiently characterized. Here, we report the fungal diversity in the pristine, oligotrophic, karst Heshang Cave, central China, using a culture-dependent method coupled with the analysis of the fungal rRNA-ITS gene sequences. A total of 194 isolates were obtained with six different media from 14 sampling sites of sediments, weathered rocks, and bat guanos. Phylogenetic analysis clustered the 194 sequenced isolates into 33 genera within 15 orders of three phyla, Ascomycota, Basidiomycota, and Zygomycota, indicating a high degree of fungal diversity in the Heshang Cave. Notably, 16 out of the 36 fungal genera were also frequently observed in solution caves around the world and 23 genera were previously found in carbonate cave, indicating potential similarities among fungal communities in cave ecosystems. However, 10 genera in this study were not reported previously in any solution caves, thus expanding our knowledge about fungal diversity in cave ecosystems. Moreover, culturable fungal diversity varied from one habitat to another within the cave, being the highest in sediments, followed by weathered rocks and bat guanos as indicated by α-diversity indexes. At the genus level, *Penicillium* accounted for 40, 54, and 52% in three habitats of sediments, weathered rocks, and bat guanos, respectively. *Trichoderma, Paecilomyces*, and *Aspergillus* accounted for 9, 22, and 37% in the above habitats, correspondingly. Despite of the dominance of *Penicillium* in all samples, β-diversity index indicated significant differences between each two fungal communities in the three habitats in view of both the composition and abundance. Our study is the first report on fungal communities in a natural pristine solution cave system in central China and sheds light on fungal diversity and functions in cave ecosystems.

## Introduction

Fungi are eukaryotic and organotrophic microorganisms, comprising at least 1.5 million species (Hawksworth, 2001), most of which are uncharacterized despite their significant roles in nature. As important decomposers of ecosystems, fungi are intimately involved in biogeochemical transformation at local and global scales and have profound influences on elemental cycling, bioweathering of rocks and minerals, and bioremediation. Fungi play an important role particularly under aerobic conditions (Sterflinger, 2000; Gadd, 2008, 2010). They are ubiquitous pioneer dwellers on surfaces of rocks (Staley et al., 1982; Sterflinger, 2000; Gorbushina et al., 2001; Etienne and Dupont, 2002) and in extremely adverse and nutrient-deprived environments including caves (Burford et al., 2003a).

Caves are dark environments with high humidity and limited temperature fluctuations. Due to the lack of organic carbon input from photosynthesis and the absence of light and various physicochemical micro-gradients, caves are considered to be extreme environments to life (Northup and Lavoie, 2001). However, high indigenous microbial diversity, unique metabolic features, and ecological functions within the domains of bacteria, archaea, and fungal groups have been observed in caves via both culture-dependent and culture-independent methods (Høeg, 1946; Caumartin, 1963; Bastian et al., 2010; Wang et al., 2010b; Vanderwolf et al., 2013; Pusz et al., 2015). The interactions between these subterranean microbes and caves have also been elucidated (Groth et al., 1999; Engel et al., 2001; Spear et al., 2007; Onac and Forti, 2011). Extensive fungal studies have been previously conducted in show caves due to the urgent need for preventing fungal colonization of frescos and other works of art of cultural heritage. It has been shown that both the number and community composition of airborne fungi are strongly correlated with numbers of cave visitors (Wang et al., 2010b) and activities of arthropods (Bastian et al., 2010; Shapiro and Pringle, 2010; Porca et al., 2011; Ogórek et al., 2013; Griffin et al., 2014).

Fungal research in pristine caves did not attract much attention until the outbreak of the lethal fungal disease, white nose syndrome (WNS), among North American bats, happened several years ago (Wibbelt et al., 2010). Since then studies concerning new drugs and novel genes have been conducted related to fungal biodiversity in pristine cave habitats (Nováková, 2009; Docampo et al., 2011; Vaughan et al., 2011; Ogórek et al., 2013; Pusz et al., 2015). Recently 1029 species in 518 genera of fungi, slime molds and fungus-like taxa have been reported (Vanderwolf et al., 2013), which filled the gap about the fungal diversity in pristine caves. Ecologically, fungi have been observed to be epi- and endolithic to various rocks including sandstone, granite, limestone, and marble (Burford et al., 2003b; Ogórek et al., 2013; Pusz et al., 2015) and even in ice caves (Tebo et al., 2015). Although fungal communities were reported to strongly influence mineral precipitation in cave environments, studies of fungal communities and their potential ecological functions in karst systems are still lacking (Gadd, 2004; Engel, 2007; Zhou et al., 2007; Wang et al., 2010b).

The Heshang Cave is a pristine carbonate cave with a slight alkalinity (pH 8.2–8.7), darkness and extremely low concentrations of mineral nutrients (Yun et al., 2015). To understand the microbially mediated geological processes in cave ecosystems, bacterial diversity (Liu et al., 2010b; Gong et al., 2015) and the role bacteria played in calcite carbonate formation and phosphate mineral dissolution have also been demonstrated (Wang et al., 2010a, 2013) in this cave. However, fungal diversity, physiology and ecological functions in cave ecosystems have yet to be characterized. Studies of cave fungi will not only expand our knowledge of microbial diversity, but will also unravel new insights into microbial ecological functions under unfavorable and nutrient-limited conditions.

Therefore the objective of this study was to investigate culturable fungal diversities in different habitats (sediments, weathered rocks and bat guanos) in the Heshang Cave via traditional cultivation techniques coupled with the analysis of the ribosome spacer sequence (ITS) gene sequencing. Our results will provide useful information about the fungal culturable techniques and valuable fungal isolates for further physiological and ecological studies.

## Materials and Methods

### Cave Description and Sampling

**FIGURE 1:**
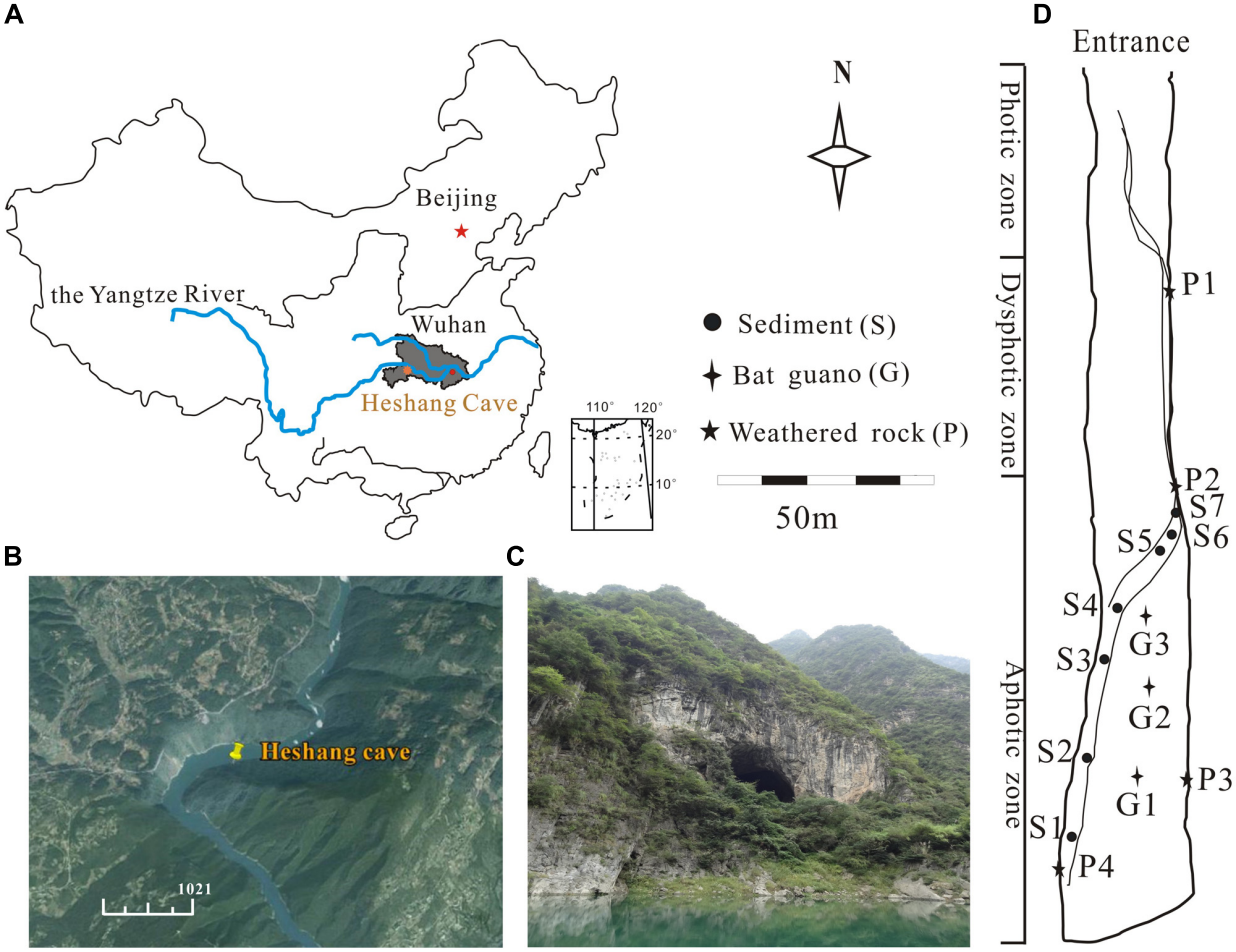
**Location of the Heshang Cave and sampling sites.** **(A)** The dark gray area shows the location of Hubei province. **(B)** The location of the Heshang Cave indicated by GPS, which lies in the south bank of the Qingjiang Valley, in the middle reaches of the Yangtze River (http://www.google.com/earth/). **(C)** Outside view of the Heshang Cave. **(D)** Sampling sites of sediments (S), weathered rocks (P), and bat guanos (G).

The Heshang Cave, a horizontally oriented solution cave, developed in Cambrian dolomite, lies in the south bank of Qingjiang Valley in the middle reaches of the Yangtze River (30°27′ N, 110°25′ E; and 294 m altitude, **Figures 1A,B**). It is an oligotrophic dark, karst cave overlain by ∼400 m of Cambrian dolomite. The cave is about 250 m long, 20 m in width and height with a sole entrance 30 m above the Qingjiang River (**Figure 1C**). The cave is only accessible by boat with seldom human disturbance. The East Asian Monsoon poses significant influence on this karst region and makes the cave wet throughout the year with an intermittent subterranean stream and active drips. The annual mean temperature is between 16 and 18°C (Hu et al., 2008).

The cave is divided into the photic, dysphotic, and aphotic zones according to the availability and intensity of light (**Figure 1D**). Surface sediments samples (<3 cm; from S1 to S7, **Figure 1D**) were collected in 1 m^2^ quadrats at 3 m intervals from the aphotic zone along the intermittent stream. Bat guano samples were taken from three guano stacking sites in the middle of the aphotic zone (**Figure 1D**). Samples of weathered rocks were collected from the dysphotic and aphotic zones (**Figure 1D**). Three composite samples were taken at each sampling site to improve the representativeness. Altogether, 42 samples from 14 sites were collected in March of 2014 with sterile spatulas and stored in 50 ml sterile plastic centrifuge tubes. The samples were transported on ice within 48 h to the Geomicrobiology Laboratory (China University of Geosciences, Wuhan) and were finely ground with a sterile mortar and pestle. Samples with the grain size of <2 mm were stored at 4°C for further processing.

### Isolation of Fungi

Six different media were employed to increase the cultivability and improve the recovery of fungi from the samples. Two oligotrophic media, corn meal agar (CMA) and sediment extract agar, [SEA; extract of 175 g sediment in 1 L distilled water and sterilized with 1.5% (w/v) agar], were used to mimic the nutrient-limited conditions in the Heshang Cave. A fungal selective medium, Martin agar (MA; per liter distilled water: 10 g glucose, 5 g peptone, 20 g agar, 0.5 g MgSO_4_⋅7H_2_O, 1.0 g KH_2_PO_4_, 3.3 ml of 1% rose-bengal solution), and three most commonly used media for isolation of cave fungi, Czapek agar (CZA), Potato dextrose agar (PDA), and Sabouraud agar (SDA), were also used (Vanderwolf et al., 2013). The composition of media CMA, CZA, PDA, and SDA is described in Atlas (2004). The initial pH of all media was adjusted to 8.0 for sediment samples and to the original pH values of weathered rocks and guano samples.

Samples (10 g) were suspended in 90 ml of 0.9% sterile saline solution and mixed thoroughly by shaking at 150 rpm for 30 min. Subsequently serial 10-fold dilutions were performed. To isolate cave-associated fungi, 200 μl aliquots of 10^-2^ and 10^-3^ dilutions of each sample were plated in triplicate onto the six media amended with penicillin and streptomycin (30 μg/ml) to inhibit bacterial growth. Sterile saline water was plated on the six different media in triplicate to serve as negative controls. All plates were incubated at 25°C for 4 weeks in the dark to allow for the development of slow-growing colonies. Fungal isolates were initially distinguished according to their phenotypic characteristics, such as color, shape, size, sclerotia, colony surface texture, hyphal pigmentation, and relative growth rates. Fungal colony forming units (CFUs) were counted and fungi with different phenotypes were isolated. Isolates were sub-cultured in PDA medium to obtain pure cultures for molecular identification. Individual pure strains were deposited in the culture collection of the Geomicrobiology Laboratory, State Key Laboratory of Biogeology and Environmental Geology, China University of Geosciences (Wuhan).

### Genomic DNA Extraction, rRNA-ITS Gene Amplification, and Sequencing

Fungal mycelia on PDA plates after 5 days of incubation at 25°C were scraped by sterile pipette tips and cells were broken with 50 μl lysis buffer [Lysis Buffer for Microorganism to Direct polymerase chain reaction (PCR), TaKaRa] in 1.5 ml micro-centrifuge tubes. Genomic DNA was extracted according to the instruction of the direct PCR (TaKaRa) method with modifications of the first step as followings: (1) incubation at 80°C coupled with oscillation for 10 min, followed by, (2) -80°C for 15 min, (3) thermal denaturation at 80°C with oscillation for 15 min, and (4) centrifugation at 5000 rpm for 5 min at 22°C. The supernatant was used as template for DNA amplification.

The ITS regions between the small subunit and large subunits of rRNA genes were amplified with the fungal specific primer ITS1 (forward; 5′-TCCGTAGGTGAACCTGCGG-3′) and universal eukaryotic primer ITS4 (reverse; 5′-TCCTCCGCTTATTGATATGC-3′; White et al., 1990; Gardes and Bruns, 1993) in 50 μl reaction mixture containing 5 μl DNA template, 25 μl Premix taq (EX Taq Version 2.0, TaKaRa), 0.5 μl of each forward and reverse primer (20 pmol/μl) and 19 μl RNA-free water (TaKaRa). The PCR amplifications were performed with a Biometra T-Gradient thermocycler (Biometra GmbH, Göttingen, Germany) using the following conditions: initial denaturing at 94°C for 10 min followed by 30 cycles (denaturation at 94°C for 30 s, annealing at 55°C for 30 s and extension at 72°C for 1 min) and a final extension at 72°C for 5 min. Negative controls (RNA-free water) were included for each set of reactions.

Polymerase chain reaction products were visualized with 1% agarose gel electrophoresis and target bands were purified using the QIA quick PCR Gel Extraction Kit (QIAGEN) according to the manufacturer’s instructions and commercially sequenced with an ABI-3730 DNA analyzer (GenScript, Nanjing, China).

### Community Diversity and Phylogenetic Analysis

All sequences were checked with the software of PlutoF workbench^1^ to remove chimeric sequences and read reliability (Nilsson et al., 2012). The fungal community comparison was analyzed at OTU level using Mothur^2^ and UniFrac with a cutoff of 1, 3, and 5% evolutionary distance respectively. The phylogenetic diversity (PD) metric for samples from sediments, weathered rocks and the bat guanos was calculated with R package (Faith, 1992). For the construction of phylogenetic tree of all culturable fungi in the cave, the representative OTUs with a cutoff of 5% were selected to avoid the diversity overestimation caused by variable ITS sequences of isolates. Sequences and the top BLAST hit in NCBI were edited and aligned using CLUSTAL-W and manually adjusted. Phylogenetic analysis was conducted using the maximum likelihood (ML) algorithm in MEGA5 (Tamura et al., 2011) based on the best-fit substitution model of nucleotide with the lowest Bayesian information criterion (BIC). Nearest neighbor interchange (NNI) of quick searches was selected as ML heuristic method. The Bootstrap analyses were run for 1,000 replicates. All the sequences in this study have been submitted to the NCBI GenBank database with accession numbers of KP734093 and KP216864 to KP217002.

## Results

### Overview of Culturable Fungi in the Heshang Cave

Altogether 194 isolates of indigenous cave fungi were obtained from all the samples using multiple types of solid media. Most of the isolates were recovered with MA (47, 24%), CZA (44, 23%), PDA (43, 22%), and SDA (39, 20%) media, indicating the applicability of these media in isolating cave fungi. A small number of isolates were recovered with CMA (15, 8%) and SEA (6, 3%) media. CFU counts on PDA in the three habitats were the highest in sediments followed by bat guanos and weathered rocks with a value of (3.6 ± 0.001) × 10^3^ CFU.g^-1^, (9.81 ± 0.07) × 10^2^ CFU.g^-1^, and (1.79 ± 0.03) × 10^2^ CFU.g^-1^, respectively.

**FIGURE 2:**
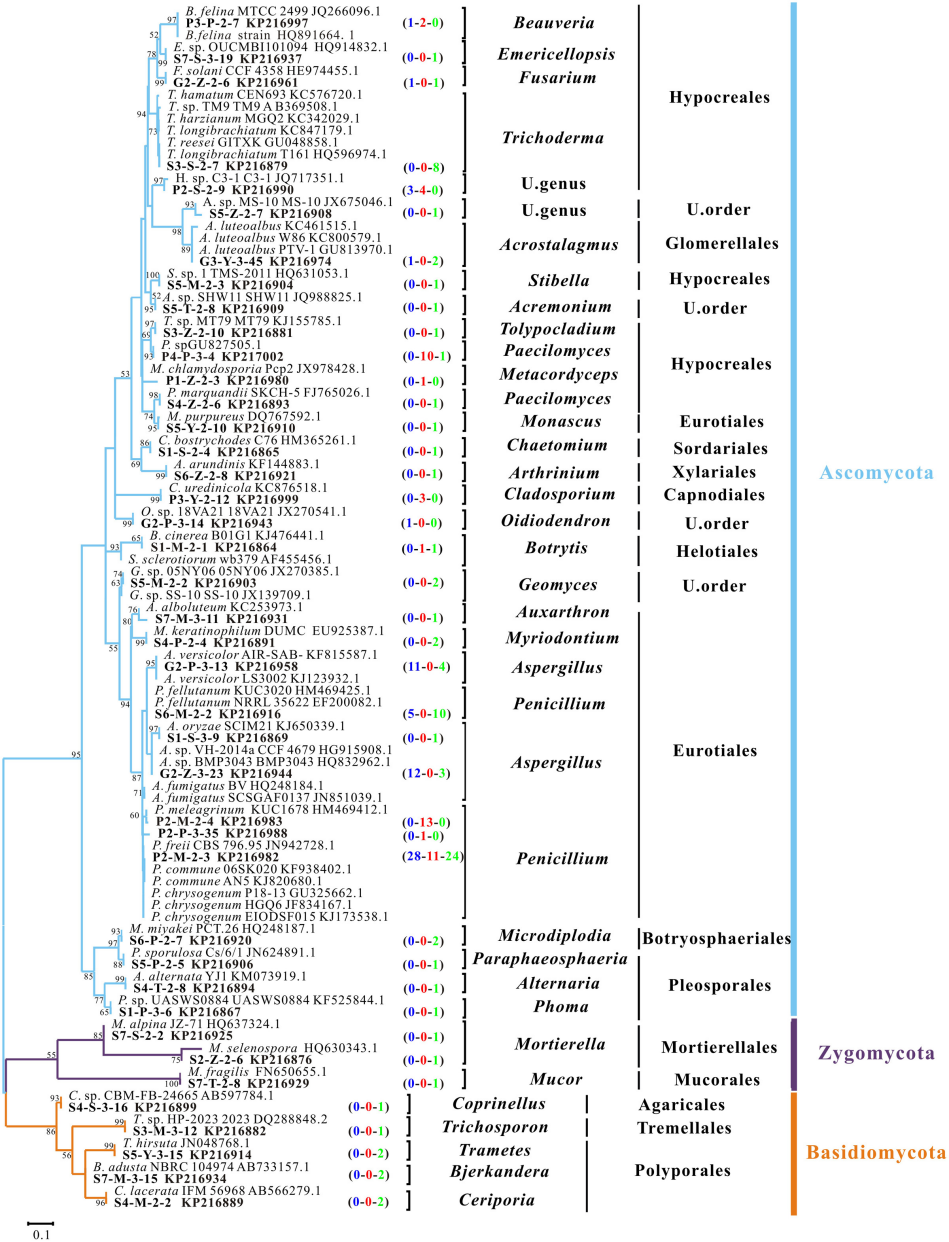
**Phylogenetic dendrogram of culturable fungal rRNA-ITS gene sequences from representative OTUs with 5% cutoff in the Heshang Cave, central China.** Maximum Likelihood algorithm with Kimura’s two parameter-Gamma distributed model; 1,000 bootstrap replicates were performed and values with >50% are shown in the tree. Sequences obtained in the present study and their GenBank accession numbers are in bold. Numbers in parenthesis indicate the sequence numbers from bat guanos (blue), weathered rocks (red), and sediments (green), respectively. U stands for unclassified.

All isolates obtained from 42 samples at 14 sampling sites were sequenced. Most sequences showed high affiliations (identity ≥ 98%) with their best matches in the NCBI database. The ITS sequences were clustered into 41 OTUs with a cutoff of 5%, which fell into 33 genera within 15 orders of three phyla (**Figure 2**). Ascomycota clearly dominated the recovered fungal community with 33 OTUs (80% of the total OTUs). In contrast, only five OTUs belonged to Basidiomycota and three to Zygomycota (**Figure 2**). At the taxonomic level of order, the culturable fungal community had nine orders and two unclassified members in Ascomycota, three in Basidiomycota and two in Zygomycota (**Figure 2**). Hypocreales (11 OTUs) and Eurotiales (10 OTUs) were the most abundant two orders (**Figure 2**). At genus level, the most frequently observed OTUs showed high affinities to *Penicillium* (**Figure 2**).

**Table 1:** Information about the 10 unique culturable fungal genera isolated from the Heshang Cave, central China.

Phylum	Order	Unique Genus	Strains	Accession No	Samples	Medium Used	Ecology	Environments Reported	Reference
Ascomycota	Hypocreales	*Metacordyceps*	P1-Z-2-3	KP216980	Weathered rocks	CZA	Keratinophilic	Deposit; birds	Hubálek et al., 2000; Vidal and Vidal, 2009
		*Stilbella*	S5-M-2-3	KP216904	Sediments	MA	Entomoparasitic	Marine; entomogenous	Jurado et al., 2008; Kafanova et al., 2008
	Pleosporales	*Paraphaeosphaeria*	S5-P-2-5	KP216906	Sediments	PDA	Saprotrophic or endophyte	Marine sponge; air	Liu et al., 2010a; Almaguer et al., 2014
	Eurotiales	*Myriodontium*	S4- P -2-4 S7- P -3-16	KP216891 KP216935	Sediments	PDA PDA	Saprotrophic	Soil	Deshmukh and Verekar, 2014
		*Auxarthron*	S7-M-3-11	KP216931	Sediments	MA	Saprotrophic	Soil	Deshmukh and Verekar, 2006
	Botryosphaeriales	*Microdiplodia*	S6-P-2-7	KP216920	Sediments	PDA	Saprotrophic or endophyte	Forest; endophytes	Siddiqui et al., 2011; Verma, 2014
Basidiomycota	Polyporales	*Trametes*	S3-T-3-18 S5-Y-3-15	KP216887 KP216914	Sediments	SEA CMA	Saprotrophic	Soil	Dhakar and Pandey, 2013
		*Bjerkandera*	S7-M-3-15	KP216934	Sediments	MA	Saprotrophic or endophyte	Compost	Taboada-Puig et al., 2011; Chen and Ting, 2015
		*Ceriporia*	S4-M-2-2 S5-Z-2-6	KP216889 KP216907	Sediments	MA CZA	Saprotrophic on wood	Wood	Suhara et al., 2003
	Tremellales	*Trichosporon*	S3-M-3-12	KP216882	Sediments	MA	Mycoses in human	Human	Colombo et al., 2011


### Diversity of Rock-inhabiting Culturable Fungi

**FIGURE 3:**
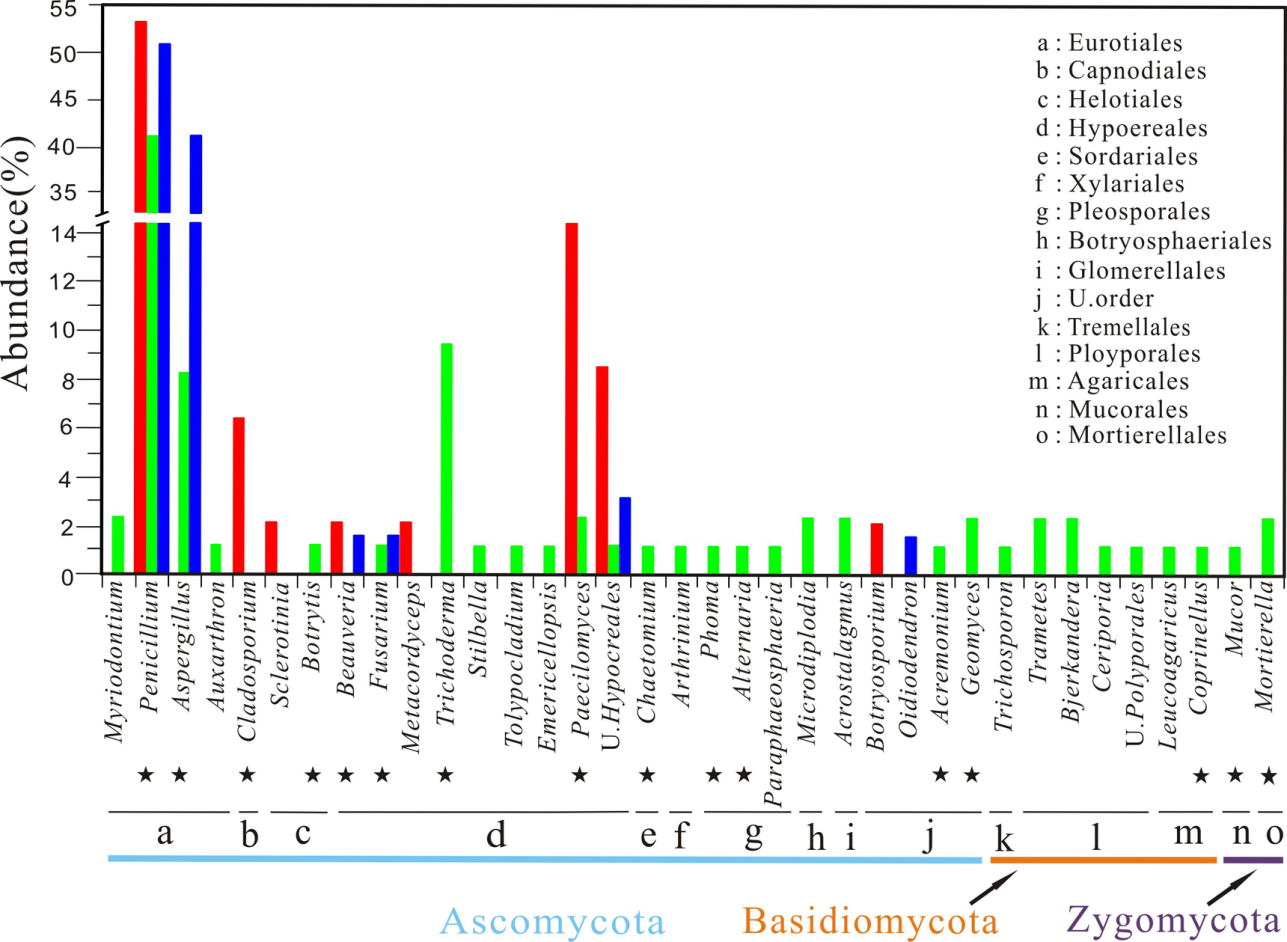
**Taxonomic distribution of cultural fungi recovered from the Heshang Cave.** Isolates from sediment, rock, and bat guano samples are marked in green, red, and blue, respectively. Commonly found genera in solution caves around the world are indicated by the black stars. Letters from a to o indicate the fungal orders detected in three habitats. U stands for unclassified.

A total of 46 pure isolates were obtained from 12 samples of weathered rocks and subjected to ITS rRNA sequencing. Phylogenetic analysis grouped these isolates into the phylum Ascomycota, corresponding to seven genera in four orders (**Figures 2** and **3**). The genera were (in the order of relative abundance) *Penicillium* (54%)*, Paecilomyces* (22%), an unclassified genus (9%), *Cladosporium* (7%), *Beauveria* (4%), *Botrytis* (2%), and *Metacordyceps* (2%). The orders included Eurotiales (54%), Hypocreales (37%), Capnodiales (7%), and Helotiales (2%; **Figure 3**). Notably, *Metacordyceps* was the unique genus present in weathered rock samples, which was not reported in solution caves around the world (**Table 1**).

Eurotiales included 25 isolates and was dominant order (relative abundance 54%) in weathered rock samples. These isolates clustered into only one well-characterized genus, *Penicillium* (**Figure 2**), which was frequently discovered in different kinds of caves (Nováková, 2009; Ogórek et al., 2013). The subordinate order Hypocreales (37%) included 17 isolates and clustered into four genera (**Figure 2**). *Paecilomyces* was relatively abundant in weathered rocks compared those in sediments and bat guanos (**Figure 2**). Two isolates formed a tight cluster closely related to the strain *Beauveria feline* HQ891664, which was a marine-derived fungus.

Generally the coverage of fungal community was over 0.87 which indicated the data can reflect the culturable fungal community in weathered rocks. The coverage increased from 0.87 to 0.89 with the increase of cutoff from 1 to 5% (**Table 2**). Meanwhile the α diversity decreased with the increase of cutoff. Chao and Shannon indexes decreased from 26 to 20, 1.91 to 1.84, respectively (**Table 2**). The PD index was 0.86, 0.80, and 0.86, respectively with a cutoff of 1, 3, and 5% (**Table 3**).

### Diversity of Cave-sediment-derived Culturable Fungi

**FIGURE 4:**
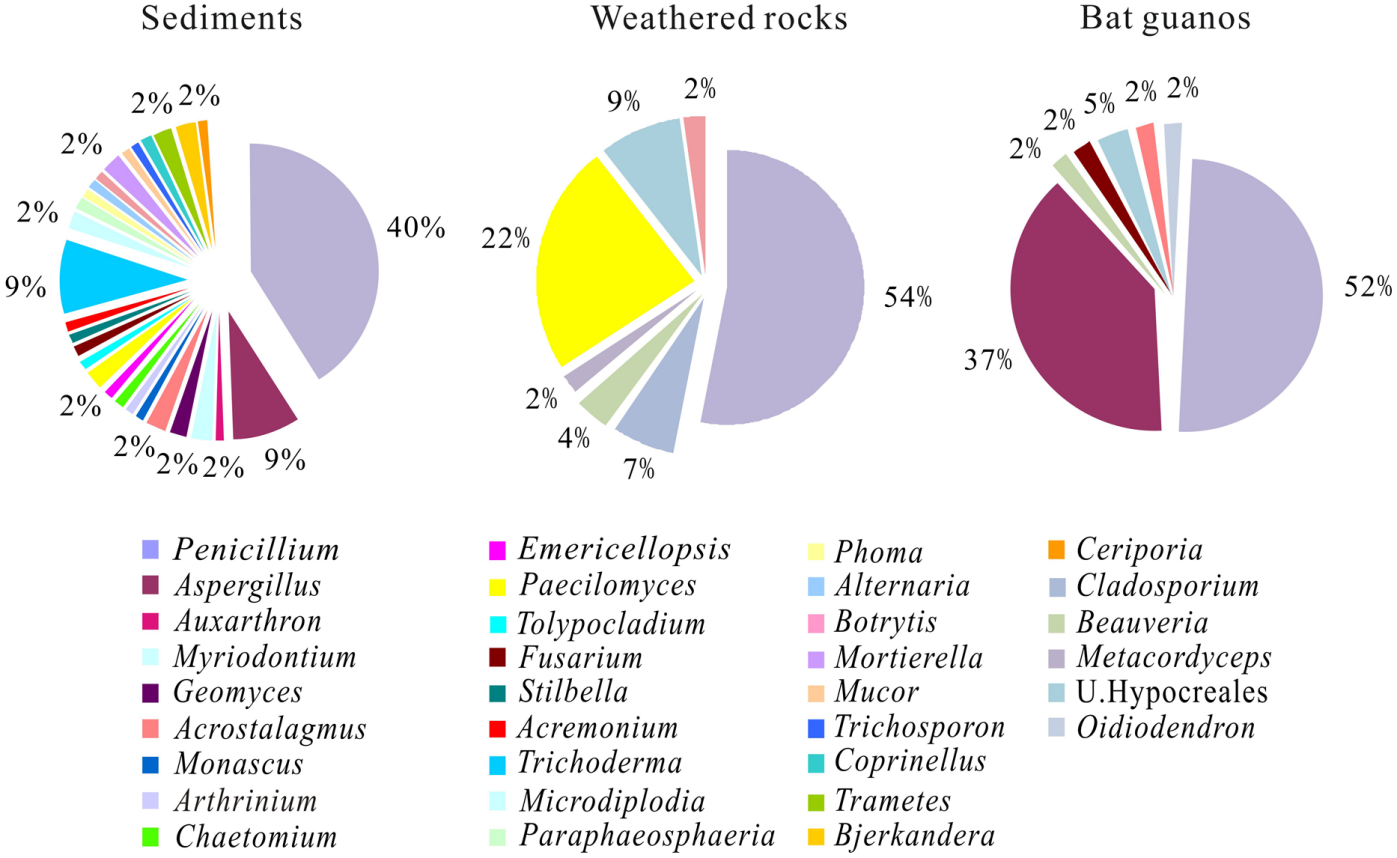
**Relative abundance of culturable fungal genus in three habitats of the Heshang Cave.** Genera with relative abundance of <2% are not shown. U stands for unclassified.

A total of 85 pure isolates were isolated from 21 samples of sediments. Phylogenetic analysis grouped these isolates into the phylum Ascomycota (87%), Basidiomycota (9%), and Zygomycota (4%, **Figure 2**), corresponding to 28 genera in 13 orders. In the order of abundance, the genera were *Penicillium*, *Aspergillus, Trichoderma, Microdiplodia*, *Mortierella*, *Acrostalagmus*, *Bjerkandera*, *Trametes*, *Ceriporia, Geomyces, Paecilomyces, Myriodontium*, *Auxarthron*, *Arthrinium*, *Chaetomium*, *Stilbella*, *Fusarium*, *Tolypocladium*, *Emericellopsis*, *Acremonium*, *Botrytis*, *Phoma*, *Alternaria*, *Mucor*, *Trichosporon*, *Coprinellus*, *Monascus*, and *Paraphaeosphaeria* (**Figure 4**). The orders included Eurotiales, Glomerellales, Sordariales, Xylariales, Hypocreales, Agaricales, Helotiales, Pleosporales, Botryosphaeriales, Mucorales, Mortierellales, Tremellales, and Polyporales (**Figure 3**). Some sporulating and ubiquitous genera such as *Aspergillus, Penicillium* were also present in sediment samples. It is noted that 13 isolates from 10 genera were not previously observed in solution caves around the world (**Figure 2**; **Table 1**). These unique isolates belonged to six orders in two phyla Ascomycota and Basidiomycota (**Table 1**).

Ascomycota was dominant and included 26 genera in 10 orders (**Figure 3**). Eurotiales (relative abundance 51%) was the most abundant order in sediment samples and included four genera of *Penicillium*, *Aspergillus*, *Myriodontium*, and *Auxarthron*. The dominant genus *Penicillium* (40%; **Figure 4**) in the order Eurotiales included 34 isolates. *Aspergillus* (relative abundance 9%) was the subordinate genus in the order of Eurotiales (**Figure 4**). Four isolates had an affiliation with *A*. *versicolor* KJ123932 and one with *A*. sp. HQ832962 (**Figure 2**), which was isolated from the bee hives and speleothem of Kartcher caverns (Vaughan et al., 2011). Hypocreales (relative abundance 18%) was the subordinate order of Ascomycota and harbored seven genera: *Trichoderma, Stilbella, Fusarium*, *Paecilomyces*, *Tolypocladium, Acremonium*, and *Emericellopsis* (**Figure 3**). Eight isolates formed a tight cluster closely related to the dominant genus *Trichoderma* (relative abundance 9%) originally isolated from soil (**Figure 2**). The other six genera in Hypocreales only harbored one isolate with relative abundance of 1% respectively (**Figures 2** and **3**).

Fungi in other orders within Ascomycota (Sordariales, Xylariales, Helotiales, Botryosphaeriales, Pleosporales) were retrieved with low relative abundance of 1–2%. Isolates in the three orders of Basidiomycota (Agaricales, Tremellales, Polyporales) and two orders of Zygomycota (Mortierellales, Mucorales) were also observed with low abundance (**Figure 3**).

The coverage of fungal community retrieved from sediments increased from 0.61 to 0.74 with the increase of cutoff from 1 to 5% (**Table 2**). Meanwhile the α diversity decreased with the increase of cutoff. Chao and Shannon indexes decreased from 104 to 63, 3.41 to 2.86, respectively (**Table 2**). The PD index was 4.00, 3.72, and 3.85, respectively with a cutoff of 1, 3, and 5% (**Table 3**).

### Diversity of Bat-guano-derived Culturable Fungi

A total of 63 isolates were retrieved from nine samples of bat guanos and all of them fell into the phylum Ascomycota. The isolates could be further affiliated with four orders and seven genera (**Figure 2**) with the dominance of Eurotiales (89%) and Hypocreales (8%). The seven genera were (in the order of abundance) *Penicillium* (52%), *Aspergillus* (37%), *Acrostalagmus* (2%), an unclassified genus (5%), *Beauveria* (2%), *Oidiodendron* (2%), and *Fusarium* (2%; **Figure 4**).

Thirty-three isolates fell into the dominant genus *Penicillium*. Five of them formed a tight cluster closely related to *P. fellutanum* (EF200082). Twenty eight strains were highly related to the *P. commune* AN5 (KJ820680), which was originally isolated in an apple orchard soil in India. *Aspergillus* was the subordinate genus and included 23 isolates. Some strains were closely related to *A. versicolor*, an opportunistic parasite in bee hives (KJ123932; Foley et al., 2014). Twelve of the isolates were clustered with *A.* sp. (HQ832962) with an identity of 100%, which was present on speleothem surface of the Kartchner Caverns (Vaughan et al., 2011).

Only several isolates fell into the orders Hypocreales (8%) and Glomerellales (2%), and which further affiliated with genera of *Beauveria*, *Oidiodendron*, an unclassified genus, *Acrostalagmus and Fusarium*. Isolate G2-P-3-14 was the only species of the genus *Oidiodendron* (relative abundance 2%) and its rRNA-ITS gene sequence was 100% identical to *Oidiodendron* sp. (JX270541) which was reported in the soil of bat hibernaculum (Lorch et al., 2013). Three strains were affiliated at identity of 100% with Hypocreaceae sp. (JQ717351) of unclassified Hypocreaceae, which was observed in corals (Xiao et al., 2011).

The coverage of fungal community was between 0.92 and 0.94 with different cutoff (**Table 2**). Shannon indexes decreased from 2.03 to 1.59 with the increase of cutoff from 1 to 5% whereas Chao showed a maximum of 21 with the cutoff of 3% (**Table 2**). The PD index (**Table 3**) showed a minimum of 0.77 at the cutoff of 3% (**Table 3**).

**Table 2:** Diversity indexes and richness metrics of culturable fungal communities within the three habitats in the Heshang Cave, central China.

Samples	Cutoff	OTUs	Chao	ACE	Shannon	Simpson	Coverage
	0.01	14	17 (14–36)	18.86	2.03	0.20	0.92
Bat guanos	0.03	11	21 (12–63)	26.32	1.74	0.25	0.92
	0.05	9	15 (10–46)	22.12	1.59	0.26	0.94
	0.01	11	26 (14–79)	49.64	1.91	0.17	0.87
Weathered rocks	0.03	10	20 (11–62)	35.44	1.84	0.18	0.89
	0.05	10	20 (11–62)	35.44	1.84	0.18	0.89
	0.01	46	104 (69–191)	263.75	3.41	0.05	0.61
Sediments	0.03	40	79 (54–143)	145.16	3.11	0.08	0.68
	0.05	34	63 (44–116)	191.55	2.86	0.10	0.74


*Numbers in parenthesis indicate the confidence intervals of Chao index*.

### Comparison of Culturable Fungal Communities

At the genus level, *Penicillium* was the most abundant and accounted for 40, 54, and 52% of cultivable fungi in the sediments, weathered rocks and bat guanos, respectively (**Figure 4**). However, subordinate genera were unevenly distributed with *Trichoderma* (9%) and *Aspergillus* (9%) in sediments, *Paecilomyces* (22%) in weathered rocks and *Aspergillus* (37%) in bat guanos respectively (**Figure 4**). The fungal composition at genus level varied among different cave niches. *Paecilomyces* was abundant in weathered rocks but seldom present in sediments. In contrast, *Trichoderma* was only present in sediments. Remarkably, sediments had more unique genera than weathered rocks, which were not previously observed in solution caves around the world (**Table 1**).

Overall OTU richness decreased with the increase of cutoff from 1 to 5% in three habitats as indicated by Chao estimator (**Table 2**). The number of OTUs decreased from 14 to 9, 11 to 10, and 46 to 34 in bat guanos, weathered rocks and sediments with the increase of cut off values respectively. The α-diversity of fungal community was relatively low in bat guanos and high in sediments as indicated by Shannon and Simpson indexes (**Table 2**). The β-diversity of fungal communities showed significant differences both in fungal composition and their abundance between each two habitats with weighted-UniFrac method despite of the different cutoff of OTUs (*P* < 0.01, **Table 4**). However, no significant differences were found in fungal community compositions between bat guanos and weathered rocks by unweighted-UniFrac analysis (**Table 4**). The PD values revealed a different pattern in three habitats of the Heshang Cave. PD was higher in sediments, and lower in weathered rocks and bat guanos (**Table 3**). Sediment samples had the largest number of OTU and corresponding highest PD value. Nevertheless, weathered rock samples had the lowest number of OTU but the PD value was higher than that of bat guanos (**Table 4**).

**Table 3:** Phylogenetic diversity (PD) metrics for culturable fungal communities with different cutoff in the three habitats of the Heshang Cave, central China.

Samples	No. of Sequences	0.01		0.03		0.05	
				
		OTU	PD	OTU	PD	OTU	PD
Bat guanos	63	14	0.79	11	0.77	9	0.83
Weathered rocks	46	11	0.86	10	0.80	10	0.86
Sediments	85	46	4.00	40	3.72	34	3.85


## Discussion

### Isolation Methods Employed

Culture-dependent methods underline their advantages in manipulating individual isolates, elucidating the physiological properties, metabolic interactions between microorganisms and the environment (Boone and Castenholz, 2001) and thus provide useful information for their potential ecological roles in ecosystems. To date, 91.5% of cave fungal studies were based on culture-dependent methods in caves (Vanderwolf et al., 2013). However, most environmental fungi are refractory to laboratory cultivation, especially many rock-dwelling fungi with low metabolic activity (Wollenzien et al., 1995). They are often overlooked within the time constraints of isolation and incubation. As for cave mycological studies, strategies of incubation such as media selection, incubation time and temperature can significantly affect cultivation results due to the different physiological requirements by different fungal taxa. Usually for SDA, CZA, and PDA media, 18∼20 days’ incubation at ≥25°C have been used for fungal isolation in solution cave and mine around the world (Vanderwolf et al., 2013). In this study, to obtain as many fungal isolates as possible, we tried multiple types of agar media besides SDA, CZA and PDA, combined with long incubation periods of 4 weeks at 25°C to investigate the diversity of culturable fungi in the Heshang Cave. The results showed that MA, CZA, PDA, and SDA media were suited to our samples in light of isolation numbers. It is noted that five media other than SDA were successfully in the recovery of unique fungal genera in this study (**Table 1**).

**Table 4:** β-diversity of culturable fungal communities between each two habitats by the weighted and unweighted Unifrac distances methods with different cutoff.

Samples	0.01		0.03		0.05	
			
	Weighted	Unweighted	Weighted	Unweighted	Weighted	Unweighted
Bat guanos – weathered rocks	0.47^∗∗^	0.69	0.47^∗∗^	0.67	0.46^∗∗^	0.65
Bat guanos – sediments	0.41^∗∗^	0.83^∗∗^	0.39^∗∗^	0.81^∗∗^	0.18^∗∗^	0.70^∗^
Weathered rocks – sediments	0.37^∗∗^	0.87^∗∗^	0.36^∗∗^	0.87^∗∗^	0.17^∗∗^	0.77^∗∗^


*^∗^P < 0.05; ^∗∗^P < 0.01.*

Interestingly, some slow-growing and pinhead-sized black colonies with melanin and convergent morphologies were visible at the end of the incubation. Some of them were covered by rapidly growing filamentous fungi and thus only can be seen from the back of Petri dishes. The observation of slow-growing colonies is consistent with what described Vanderwolf et al. (2013) previously. Usually it will take >30 days for slow-growing fungi to form visible colonies due to their low metabolism rates or refractory to laboratory cultivation or present in low cell numbers. Therefore, an appropriate length of incubation should be considered to allow slow-growing fungi to develop colonies in cave fungal diversity investigation.

### Culturable Fungal Diversity

To date, 36 genera have been most frequently isolated from solution caves and mines around the world (Vanderwolf et al., 2013). Among them 16 (**Figure 3**) were also isolated in this study from the Heshang Cave, which indicates a highly diverse fungal community in our cave and the high efficiency of our culture-dependent methods. Moreover, we found 23 out of 33 genera in this study were previously reported in carbonate caves (Nováková, 2009; Ogórek et al., 2013; Vanderwolf et al., 2013), indicating the potential similarities among fungal communities in karst cave ecosystems around the world (**Figure 3**).

At the phylum level, Ascomycota dominated the recovered community in this study, which is consistent with the statistical results of fungal community composition in caves and mines reported previously with Ascomycota (69%) as the dominant phylum (Vanderwolf et al., 2013). In contrast, members in Basidiomycota were difficult to culture and strongly favor for nutrient rich substrates such as dung in caves (Vanderwolf et al., 2013). Thus the oligotrophic conditions in the Heshang Cave may be adverse to their growth and result in lower numbers of isolates.

At genus level, 33 genera were observed and only five of them were shared by two habitats; others were exclusively present in one specific habitat. Fungal diversity was the highest in sediments, followed by weathered rocks and bat guanos as indicated by α-diversity indexes (**Table 2**). These results were consistent with the results of fungal communities in caves around the world (Vaughan et al., 2008; Vanderwolf et al., 2013).

Moreover β-diversity also indicated significant differences between the fungal communities in each two habitats in view of the composition and abundance of fungal communities (**Table 4**). These results revealed a highly diverse fungal world in different habitats of the Heshang Cave and have expanded our knowledge about fungal diversity in cave ecosystems. Nonetheless, the diversity in three habitats in the Heshang Cave is likely to be higher due to the reasons of (i) the limited samples in this study, (ii) culture method employed, and (iii) low curability of microbes in nature.

### Unique Genera in this Study

The isolation of 194 strains and in particular the 10 unique genera in this study provided new information about culturable fungal taxonomic diversity in solution caves. To our knowledge, these 10 unique fungal genera have yet to be reported in solution caves around the world to date. Among them, six genera belong to Ascomycota and four to Basidiomycota, accounting for 60 and 40% of these unique groups respectively (**Table 1**). These unique genera displayed low isolation frequency and only accounted for 1–2% of total isolates within samples. Only *Metacordyceps* was present in weathered rocks. In contrast, the other nine genera were exclusively present in sediments (**Table 1**). These genera have been found previously in other environments such as soil, marine, air, forest, compost, deposit, mammals, birds and plant endophytes (**Table 1**). However the physiology and ecology of these unique genera still remain poorly understood at regional and global scales in solution caves, which merits further investigation.

## Conclusion

The diversities of the fungal communities associated with the three habitats in the Heshang Cave were investigated by the culture-dependent method together with the analysis of the fungal rRNA-ITS gene sequences. Fungal communities showed significant differences in consideration of composition and abundance among different cave niches although some similarities in the taxonomic structures were observed. Our results, especially the unique genera unreported in solution caves previously, provide valuable information on cave-associated culturable fungal diversity. This is the first report on fungal communities in a natural pristine solution cave system in central China to our knowledge and sheds light on fungal diversity and functions in cave ecosystems.

## Author Contributions

BM carried the fungal isolation and identification work and prepared the manuscript draft. HW provided the research idea and funding for this study and improved the scientific and technical content of manuscript. XX, YY, and LG assisted with the phylogenetic analysis. RW helped with the fungal isolation and cultivation work.

## Conflict of Interest Statement

The authors declare that the research was conducted in the absence of any commercial or financial relationships that could be construed as a potential conflict of interest.

## Footnotes

1 http://plutof.ut.ee

2 http://www.mothur.org
